# Draft Genome Sequence of *Burkholderia* sp. Strain PML1(12), an Ectomycorrhizosphere-Inhabiting Bacterium with Effective Mineral-Weathering Ability

**DOI:** 10.1128/genomeA.00798-15

**Published:** 2015-07-23

**Authors:** Stéphane Uroz, Phil Oger

**Affiliations:** a INRA, UMR 1136 INRA, Université de Lorraine Interactions Arbres Micro-organismes, Centre INRA de Nancy, Champenoux, France; b INRA UR 1138 Biogéochimie des Ecosystèmes Forestiers, Centre INRA de Nancy, Champenoux, France; c CNRS UMR 5276, ENS de Lyon, Laboratoire de Géologie de Lyon, Lyon, France

## Abstract

We report the draft genome sequence of *Burkholderia* sp. PML1(12), a soil bacterium isolated from the Oak-*Scleroderma citrinum* ectomycorrhizosphere in the experimental forest site of Breuil-Chenue (France).

## GENOME ANNOUNCEMENT

Minerals represent the main source of nutritive cations in acidic and nutrient-poor forest soils in temperate regions (1). However, the nutrients entrapped in their structure are not directly accessible to the tree roots, which rely on soil microorganisms to weather the minerals (2, 3). Functional screening of bacterial strains isolated from the *Scleroderma citrinum* ectomycorrhizosphere and the surrounding bulk soil revealed an enrichment of effective mineral-weathering bacteria in the tree root vicinity (4–7). Notably, cultivation-dependent and -independent approaches revealed that the *Burkholderia* genus was the most abundant and the most effective mineral-weathering genus (6–9). A significant positive correlation was observed between the abundance of *Burkholderia* on minerals incubated in forest soils and the weathering level of these minerals (9, 10). Among *Burkholderia* strains tested, strain PML1(12) appeared the most effective and capable of improving tree growth in nutrient-poor condition microcoms (11). With the goal of characterizing this strain and of identifying the molecular mechanisms involved in its mineral-weathering ability, we determined the complete sequence of its genome.

Genomic DNA was obtained using the protocol of Pospiech and Neumann (12). The genomic DNA was sequenced on a genome sequencer (GS) FLX 454 System (Roche) at Cogenics (Meylan, France). A half-run was used to perform 3-kb paired-end tags (L-PET) yielding 1,452,141 reads that were 385 bp long on average. A *de novo* assembly was performed using Newbler 2.6. The draft genome has 293 contigs larger than 500 bp, which were assembled in 8 scaffolds, with a calculated total length of 9,340,894 bp (35× average depth of coverage), a G+C content of 60.83%, and an *N*_50_ contig size of 70,828 bp. The largest contig generated was 272,460 bp long. We aligned the draft genome with the 10 closest neighboring complete genomes (chromosomes and plasmids) of the *Burkholderia* genus using average nucleotide identity (ANI) tools (http://enve-omics.ce.gatech.edu/ani/) (13). The best ANI score (80.83% average nucleotide identity) was obtained with *Burkholderia glathei* strain LMG14190T, indicating that strain PML1(12) could represent a novel species within the *Burkholderia* genus.

According to the automated annotation done using RAST, the genome contains a total of 8,761 predicted protein-coding genes, from which 2,241 (25.6%) were annotated as encoding hypothetical proteins. The genome contains a total of 46 tRNA coding genes. In addition, the genome of strain PML1(12) contains at least 4 complete copies of ribosomal operons of the following structure: 5S/23S/tRNA-ala-TGC/tRNAile-GAT/16S. The genome of strain PML1(12) presents genes related to plant growth promotion, including the genes for the production of indole-3-acetic acid (accD). The genome presents one *N*-acylhomoserine lactone quorum-sensing system. Interestingly, the PML1(12) genome shows a large number of genes (690) for the metabolism of sugars and carbohydrates, including genes for the synthesis and transport of gluconate, an organic acid for which production has been previously linked with the ability of bacteria to weather minerals. The genome possesses 15 genes annotated as siderophore transporters, which indicates the ability of the strain to scavenge Fe-loaded siderophores synthesized by other bacteria.

### Nucleotide sequence accession number.

This whole-genome shotgun project has been deposited at DDBJ/EMBL/GenBank under the accession no. AEJF00000000 (project ID PRJNA53985; sample ID 53985).

## References

[1] Uroz S, Oger P, Lepleux C, Collignon C, Frey-Klett P, Turpault MP 2011 Bacterial weathering and its contribution to nutrient cycling in temperate forest ecosystems. Res Microbiol 162:820–831. doi:10.1016/j.resmic.2011.01.013. 21315149

[2] Landeweert R, Hoffland E, Finlay RD, Kuyper TW, van Breemen N 2001 Linking plants to rocks: ectomycorrhizal fungi mobilize nutrients from minerals. Trends Ecol Evol 16:248–253. doi:10.1016/S0169-5347(01)02122-X. 11301154

[3] Uroz S, Calvaruso C, Turpault MP, Frey-Klett P 2009 Mineral weathering by bacteria: ecology, actors and mechanisms. Trends Microbiol 17:378–387. doi:10.1016/j.tim.2009.05.004. 19660952

[4] Calvaruso C, Turpault MP, Leclerc E, Ranger J, Garbaye J, Uroz S, Frey-Klett P 2010 Influence of forest trees on the distribution of mineral weathering-associated bacterial communities of the *Scleroderma citrinum* mycorrhizosphere. Appl Environ Microbiol 76:4780–4787. doi:10.1128/AEM.03040-09. 20511429 PMC2901721

[5] Uroz S, Calvaruso C, Turpault MP, Pierrat JC, Mustin C, Frey-Klett P 2007 Effect of the mycorrhizosphere on the genotypic and metabolic diversity of the bacterial communities involved in mineral weathering in a forest soil. Appl Environ Microbiol 73:3019–3027. doi:10.1128/AEM.00121-07. 17351101 PMC1892860

[6] Uroz S, Buée M, Murat C, Frey-Klett P, Martin F 2010 Pyrosequencing reveals a contrasted bacterial diversity between oak rhizosphere and surrounding soil. Environ Microbiol Rep 2:281–288. doi:10.1111/j.1758-2229.2009.00117.x. 23766079

[7] Uroz S, Tech JJ, Sawaya NA, Frey-Klett P, Leveau JHJ 2014 Structure and function of bacterial communities in ageing soils: insights from the Mendocino ecological staircase. Soil Biol Biochem 69:265–274. doi:10.1016/j.soilbio.2013.11.002.

[8] Collignon C, Uroz S, Turpault M, Frey-Klett P 2011 Seasons differently impact the structure of mineral weathering bacterial communities in beech and spruce stands. Soil Biol Biochem 43:2012–2022. doi:10.1016/j.soilbio.2011.05.008.

[9] Lepleux C, Turpault M-P, Oger P, Frey-Klett P, Uroz S 2012 Correlation of the abundance of betaproteobacteria on mineral surfaces with mineral weathering in forest soils. Appl Environ Microbiol 78:7114–7119. doi:10.1128/AEM.00996-12. 22798365 PMC3457497

[10] Uroz S, Turpault MP, Delaruelle C, Mareschal L, Pierrat J-, Frey-Klett P 2012 Minerals affect the specific diversity of forest soil bacterial communities. Geomicrobiol J 29:88–98. doi:10.1080/01490451.2010.523764.

[11] Calvaruso C, Turpault M-P, Frey-Klett P 2006 Root-associated bacteria contribute to mineral weathering and to mineral nutrition in trees: a budgeting analysis. Appl Environ Microbiol 72:1258–1266. doi:10.1128/AEM.72.2.1258-1266.2006. 16461674 PMC1392890

[12] Pospiech A, Neumann B 1995 A versatile quick-prep of genomic DNA from Gram-positive bacteria. Trends Genet 11:217–218. doi:10.1016/S0168-9525(00)89052-6. 7638902

[13] Goris J, Konstantinidis KT, Klappenbach JA, Coenye T, Vandamme P, Tiedje JM 2007 DNA-DNA hybridization values and their relationship to whole-genome sequence similarities. Int J Syst Evol Microbiol 57:81–91. doi:10.1099/ijs.0.64483-0. 17220447

